# Temporal habitat partitioning and resource competition between congenerics: Testing for density-dependent growth and mortality in estuarine shrimp

**DOI:** 10.1371/journal.pone.0316219

**Published:** 2024-12-27

**Authors:** Robert P. Dunn, Matthew E. Kimball, Bruce W. Pfirrmann, Andrew S. Bruck, Willa M. Lane

**Affiliations:** 1 North Inlet-Winyah Bay National Estuarine Research Reserve, Georgetown, SC, United States of America; 2 Baruch Marine Field Laboratory, University of South Carolina, Georgetown, SC, United States of America; 3 Department of Environmental Studies, Cornell College, Mount Vernon, IA, United States of America; 4 School of Marine Science and Policy, University of Delaware, Lewes, DE, United States of America; Texas A&M University, UNITED STATES OF AMERICA

## Abstract

Habitat partitioning can promote coexistence of closely related competitors. Two congeneric shrimps (brown shrimp, *Penaeus aztecus*, and white shrimp, *Penaeus setiferus*) which utilize estuaries in the southeastern U.S. temporally partition much of their nursery habitat occupancy but also experience a period of overlap at the respective tails of their nursery residency. Throughout nursery residency, when conspecific or congeneric abundance can be high, density-dependent (D-D) processes may alter demographic rates, but the relative importance of the potential biotic interactions occurring in these habitats (e.g., intra- and inter-specific competition, cannibalism, among others) remains underexplored. Here, we documented the periods of nursery habitat use for these two penaeid shrimp species within a representative salt marsh estuary. Next, a set of manipulative laboratory experiments was conducted to test if conspecific or congeneric density, as well as the relative proportions of each species, affect growth and mortality. In three experiments designed to simulate each of the phases of penaeid shrimp nursery habitat use (brown only, brown and white overlap, white only), shrimp were maintained at ecologically relevant densities (12.5–37.5 m^-2^) and tagged to follow growth trajectories. We found varying degrees of density-dependence between species and across response variables (length, mass, mortality), with the effect of additional species identity varying between white and brown shrimp for all three response metrics. Body size was an important predictor of mortality for both brown and white shrimp, with smaller animals exhibiting higher mortality probabilities. These results suggest that changing environmental conditions could lead to D-D impacts on some demographic rates but not others for these ecologically and economically important species during their estuarine residency.

## Introduction

Intra- and interspecific interactions are key determinants of community composition and dynamics. In both cases, the densities of animals, whether conspecifics or other species in a community, can drive individual and population-level responses, including impacts to individual growth rates and body condition [1,2], population growth rates [3], population size [4], and behavior [5]. Negative density-dependence (or simply density-dependence) occurs when a vital rate changes with increasing organismal density based on a biological feedback [6], and can occur at the population level (e.g., reduced recruitment to saturated habitats) or within individuals. Competition for limited resources is a typical means by which density-dependence can act on individuals by, for example, slowing somatic growth at high densities [7]. Within a species, the potential for density-dependence can be reduced through partitioning whereby individuals utilize a variety of resources [8] or occupy different habitats over a complex life cycle [9]. Similarly, temporal [10] and spatial [11] habitat partitioning can promote coexistence of closely related competing species, and the level of competition can determine the degree of overlap [12,13]. Despite the fundamental nature of these processes, the relative importance of density-dependence, intra-, and inter-specific interactions are not often considered simultaneously.

Estuaries serve as critical nursery habitat for a diverse set of economically important fish and shellfish [14], and estuaries at temperate latitudes are characterized by distinct seasonality where nursery function is temporally restricted. Two species of closely related decapod crustaceans, white shrimp (*Penaeus setiferus*, formerly *Litopenaeus*) and brown shrimp (*Penaeus aztecus*, formerly *Farfantepenaeus*), reside in estuaries of the southeast United States and Gulf of Mexico during multiple life stages. Both species enter through estuarine inlets as postlarvae and subsequently use marsh surface, marsh edge, and tidal creek habitats until they migrate to deeper inshore open water habitats and ultimately back offshore as spawning adults. Both brown and white shrimp are annual species which can be highly abundant within their native range [15–17], but also exhibit substantial inter-annual variability in abundance [18]. In addition, these species support lucrative fisheries and appear to temporally partition their use of estuarine nurseries. Based on historical collections from the 1980s through 1990s in tidal creek habitats in North Inlet Estuary in South Carolina USA, juvenile brown shrimp began appearing in early spring (April), with a peak in abundance during the late spring and early summer period from May–June [19] before migrating to deeper, open water inshore habitats [20]. Juvenile white shrimp began occupying these habitats in early summer, exhibited a peak in abundance in late summer (August) and had a more prolonged estuarine residency period, through the late fall [19]. Thus, juvenile brown and white shrimp historically displayed a period of maximum overlap in tidal creek habitats during mid-summer (June and July). However, these species also exhibit substantial inter-annual variability in recruitment to estuarine nursery habitats, with juvenile abundance ranging across at least two orders of magnitude [18]. These ‘good’ and ‘bad’ juvenile recruitment years are potentially driven by abiotic forcing during the larval and postlarval stages, such that biotic interactions among juveniles and sub-adults may differ depending on the level of recruitment success into estuaries. Additionally, in recent decades we have observed considerable environmental change in our focal study system, the North Inlet Estuary. For example, subtidal creeks in this system have warmed during the winter and summer periods, while salinities during summer and fall have been reduced [21]; chl-*a* concentration in surface waters increased beginning in the mid-2010s [22]; and tidally-driven submergence time has increased, modifying nutrient dynamics on the intertidal marsh platform [23]. Each of these changes may independently or synergistically impact shrimp populations in species-specific ways, demonstrating the need to update our understanding of the context-dependency of biotic interactions between these species.

Despite their close phylogenetic relationship [24] and relatively similar life history, these species appear to differ in their sensitivity to environmental conditions. This includes a contrasting tolerance of temperature extremes with brown shrimp less tolerant of warmer water temperatures and white shrimp more susceptible to colder temperatures [17,25–28]. Brown shrimp also exhibit a distinct salinity preference (17–35 psu, and avoidance of extreme lows) whereas white shrimp do not [29]. Similarly, white and brown shrimp differ in their responses to marsh landscape features, with white shrimp size negatively correlated to patch shape complexity while brown shrimp size is positively correlated with this metric [30]. Considering these differences in habitat utilization, natural or anthropogenically driven variation in environmental conditions may lead to species-specific patterns in shrimp density through space and time. These emergent species-specific patterns may, in turn, result in increased spatial or temporal overlap between species during their nursery residency, potentially undercutting their adaptive mechanisms of habitat (resource) partitioning.

Here, we first seek to verify the hypothesis that brown and white shrimp temporally partition their use of tidal creek nursery habitats by quantifying the relative abundances and size distribution of each species based on high-frequency field sampling. Additionally, we aim to quantify the contemporary degree of temporal overlap in tidal creek nursery habitat between the two species. We then investigate the potential for impacts to growth and mortality of brown and white shrimp under different nursery conditions using a series of manipulative laboratory experiments. These experiments approximate the ‘good’ and ‘bad’ juvenile recruitment years that characterize these species population dynamics. We hypothesized that growth and mortality would exhibit negative density-dependence for both shrimp species when found in monoculture, and that the presumed inferior competitor in warmer mid-to-late summer conditions (brown shrimp) would exhibit similar density-dependent responses during the period of estuarine habitat overlap by the two species. Our field sampling and manipulative experiments contribute to improved understanding of the processes governing spatial and temporal patterns in habitat utilization within estuarine nurseries for penaeid shrimps and other nekton with similar life history strategies.

## Materials & methods

### Field sampling

To put our experimental treatments into context, we first characterized the temporal habitat utilization periods for penaeid shrimp within intertidal creeks. We sampled brown and white shrimp in Oyster Landing Creek, an intertidal salt marsh creek bordered by smooth cordgrass *Spartina alterniflora* within the North Inlet estuary (33°21′ 02.8 N, 79°11′ 27.4 W) in Georgetown County, South Carolina, USA. As part of an ongoing research program [19], seining occurred within an hour of low tide approximately every two weeks from April–September in 2022 and again in 2023. During each sampling event, we hauled a bag seine (15.2 m long, 1.2 m high, 6 mm mesh) a fixed length (~ 50 m) along the creek center channel axis. Following retrieval of the seine, all collected individuals were identified to species and counted. In addition, we measured cephalothorax length (RCL; tip of rostrum to posterior of carapace; [31]) for up to 30 individuals of each species. We converted shrimp RCL measurements into total length (TL) using length conversions derived from high-frequency shrimp collections conducted in the spring, summer, and fall of 2021 [32]. Additional details for this study site can be found in Kimball et al. [19].

### Experimental overview

We conducted manipulative experiments within a flow-through seawater system at the University of South Carolina’s Baruch Marine Field Laboratory (BMFL) during the summer months (June–August) of 2022 and 2023. During 2022, we ran trials which investigated the potential for density-dependent (D-D) growth and mortality for brown (*Penaeus aztecus*) and white (*Penaeus setiferus*) shrimp when found in monoculture. The brown shrimp trial occurred first, during the period when brown shrimp are the dominant species observed within tidal creeks (24 June– 9 July 2022). Following this, the white shrimp trial occurred during the period when white shrimp are dominant (21 July– 5 August 2022). During 2023, we conducted a trial to investigate the influence of density and relative abundance of each species during the period when both species are commonly found within estuaries (hereafter the ‘overlap’ experiment; 27 June– 12 July 2023).

We monitored water quality daily in each tank using a handheld multiparameter instrument (Yellow Springs Instruments, Inc.) to measure water temperature, salinity, and dissolved oxygen (DO). Due to the flow-through nature of our set up in the BMFL outdoor seawater laboratory, temperature, salinity, and DO in experimental arenas reflect in situ conditions. Estuaries are variable ecosystems such that the environmental conditions present in our arenas during each of the three experimental trials were well within the long-term range experienced by both shrimp species at our study site and are not considered biologically stressful for these organisms (Table 1, S1 Fig in S1 File). Thus, we are confident in comparing shrimp growth and mortality across trials and do not attribute any observed differences to the conditions within experimental arenas.

**Table 1:** Experimental treatments and conditions.

Species	Dates	Treatment	Initial Length (mm TL) with SE	Initial Wet Mass (g) with SE	Temp. (° C) with SE	Salinity (psu) with SE	DO (mg L^-1^) with SE
*Penaeus aztecus* (brown shrimp)	24 June - 9 July, 2022	Low density: Medium density: High density:	71.4 (1.73) 71.2 (1.30) 74.0 (0.95)	3.0 (0.18) 2.9 (0.14) 3.2 (0.12)	27.3 (0.04)	34.5 (0.09)	5.2 (0.03)
*Penaeus setiferus* (white shrimp)	21 July - 5 August, 2022	Low density: Medium density: High density:	74.8 (1.48) 76.9 (1.15) 75.6 (0.79)	3.3 (0.20) 3.4 (0.17) 3.2 (0.11)	28.2 (0.02)	33.0 (0.07)	5.9 (0.02)
*Penaeus aztecus* & *Penaeus setiferus*	27 June - 12 July, 2023	Medium density (1 B, 1 W): High density (2 B, 1 W): High density (1 B, 2 W):	B: 70.1 (2.79) W: 69.3 (2.4) B: 73.1 (1.88) W: 70.0 (2.11) B: 73.6 (2.31) W: 66.3 (1.52)	2.7 (0.36) 2.3 (0.24) 3.0 (0.23) 2.5 (0.24) 3.1 (0.26) 2.1 (0.13)	27.5 (0.04)	33.5 (0.07)	5.9 (0.03)

Start (Day 0) and end (Day 15) dates for monospecific (summer 2022) and overlap (summer 2023) experiments, shrimp length and mass (mean mm total length and mean wet mass in grams with standard error, SE) on Day 0 across treatment groups (B = brown shrimp, W = white shrimp), and environmental conditions (mean values with SE for water temperature, salinity, and dissolved oxygen) within mesocosms during the penaeid shrimp seawater laboratory experiments. Low, medium, and high density treatments represent 1, 2, and 3 shrimp per tank, respectively, and correspond to densities of 12.5, 25, and 37.5 shrimp m^-2^.

### Experimental setup

For all trials, we collected juvenile shrimp from tidal creeks within the North Inlet estuary using cast nets, kick nets, and bag seines. Following capture, animals were transported to the BMFL outdoor seawater laboratory. Prior to use in experiments, shrimp were kept for at least 24 h in holding tanks (284 L) continuously supplied with ambient seawater from the adjacent Crab Haul creek (i.e., a flow-through seawater system). Shrimp were offered pelletized fish feed, dried krill, and frozen grass shrimp *ad libitum* while in holding tanks. Prior to each monoculture experiment, we injected every shrimp with a unique passive integrated transponder (PIT) tag in order to identify each animal for growth measurements, maintain experimental density treatments, and quantify individual mortality likelihood. For the overlap experiment, which included both species, PIT tags were unnecessary and instead we used tailfin clipping [33] to identify shrimp. Neither PIT tags nor tailfin clipping led to changes in growth or enhanced mortality rates compared to control (untagged or unclipped) animals [34] (S1 Table in S1 File, S2 Fig in S1 File).

Each trial lasted 15 d and utilized 36 round mesocosms (bottom area = 0.08 m^2^) filled to a depth of 0.2 m with aerated raw seawater through continuously flowing individual water lines. Mesocosms were checked multiple times daily and we used a siphon to clear debris from the bottom of each mesocosm at least once every three days. For the brown and white shrimp monoculture experiments, we maintained shrimp at low (n = 1 individual), medium (n = 2), and high (n = 3) density treatment levels corresponding to 12.5, 25, and 37.5 individuals m^-2^, respectively. For the overlap experiment, we created three treatments which varied in both density and relative abundance of each species. A medium density treatment (25 m^-2^) included brown and white shrimp in equal proportion (n = 1 individual of each), while two high density treatments (37.5 m^-2^) were made up of either twice as many brown as white shrimp or twice as many white as brown shrimp (n = 1 for one species and n = 2 for the other). In all cases, these densities of juvenile penaeid shrimp have been previously observed within our system [18,35] and other southeastern US estuaries [17,36]. At the outset of their respective monoculture trials, experimental brown and white shrimp were similar in size (Table 1), with TL and mass ranging from 61–90 mm and 1.9–5.9 g, respectively. Conversely, during the overlap experiment, brown shrimp (TL range: 53–89 mm, mass range: 1.1–5.6 g) were generally larger than white shrimp (TL range: 51–83 mm, mass range: 0.8–3.9 g; Table 1), as expected based on their recruitment timing.

On days 0, 5, 10, and 15 in each experiment, we collected three measurements on every shrimp: total length in mm (TL, tip of the rostrum to tip of the telson), carapace length in mm (CL, anterior and posterior margins of carapace), and wet mass (g). For PIT-tagged shrimp, mass values were corrected to remove the weight of the tag. Total length measurements for shrimp with a broken rostrum were estimated using species-specific conversion relationships. Shrimp were checked at least 3 times daily throughout each experiment for mortalities. When we observed a dead shrimp, it was identified via its PIT tag ID or tailfin clip, then immediately replaced with a new individual which was measured and weighed just prior to being introduced. This was done to maintain density treatments but was also based on the assumption of an open population for these animals during their nursery habitat period (i.e., compensatory recruitment into tidal creeks can replace individuals that die). We believe this is justified because recruitment into tidal creeks occurs continuously during our focal study period [20], such that our strategy to replace dead animals during the experimental trials approximates the dynamics occurring during ‘good’ or ‘bad’ recruitment years.

We created individual food rations for each tank, and this same ration was used across all experiments and treatments. Specifically, seawater, grass shrimp (*Palaemonetes* spp.), pelletized fish feed, and spirulina algae powder [37] were combined in a standard food processor in a ratio of 12:5:5:1. This was blended into a slurry, and we then used a large gauge syringe to create individual 1.0 ± 0.05 g food rations using an ice cube tray placed on top of a balance. Food rations were frozen and then a single ration was placed into each tank once daily. We provided this amount of food based on the average mass of individual experimental shrimp (~ 3 g). This resulted in an average food allotment of ~ 10–30% of body mass daily (depending on density treatment), which is in line with standard feed rations used in shrimp aquaculture applications [38].

### Data analysis

We examined the temporal habitat utilization periods of brown and white shrimp in Oyster Landing Creek by investigating trends in shrimp abundance and size across sampling months. Biweekly catch values (number of individuals per seine haul) were averaged by month and year (April–September, 2022 and 2023,; number of seine hauls per monthly mean value = 1–3) for visualization. We then constructed monthly length frequency histograms for both species for each year (2022 and 2023), using shrimp total length (TL, mm) as the response variable. For months of identified overlap between brown and white shrimp, we evaluated whether the relative abundance (number of individuals per seine haul) differed between species using non-parametric Mann-Whitney U-tests. Due to relatively low monthly sample size, we pooled catch data across months of overlap and performed separate tests for each year. Similarly, we compared length-frequency distributions between brown and white shrimp for months of overlap by means of non-parametric Kolmogorov-Smirnov (K-S) tests. We performed separate K-S tests for each year and month of overlap.

Utilizing data from our three experimental trials, we investigated the species-specific impacts of shrimp density and identity of co-occurring shrimp on individual growth and mortality. Like other decapod crustaceans, shrimp grow via ecdysis (molting) and thus can only obtain a longer body size following a molt. Conversely, shrimp can exhibit slight changes in mass of soft tissues during the intra-molt period. Thus, we calculated two metrics to characterize shrimp growth over the course of the experiment: instantaneous growth (% d^-1^) in terms of both length (TL) and mass. These were calculated as [ln(*F*/*I*)/*t*]*100, where *F* is the final measurement made on a given animal, *I* is the measurement made at their introduction to the experimental mesocosm, and *t* is time spent in the experiment. Only shrimp that were alive for at least 5 days were included in these calculations (n = 75 for brown and n = 82 for white shrimp in monoculture; n = 49 brown and 50 white shrimp in the overlap experiment). We observed molting during the experiments, so expected changes in both shrimp length and mass. Estimates of per capita mortality were calculated only for the original animals included at the outset of each experiment (n = 72 in both monoculture trials, n = 96 in the overlap trial), and each animal was coded as either alive (= 0) or dead (= 1) at the end of the 15 d trials.

We statistically compared mean instantaneous growth in terms of mass and length, as well as per capita mortality, using a series of ANOVA models. Models included the same set of predictors for each of the three response variables, and were formulated as

y∼Species+Density+Identity+Species*Density+Species*Identity,

where *y* represents any of our three response variables (instantaneous growth in mass, instantaneous growth in length, mortality), *Species* identifies the focal shrimp (brown or white), *Density* reflects the number of additional shrimp in the tank with the focal shrimp (0, 1 or 2), and *Identity* codes the make-up of the additional shrimp (none, both species, brown, or white). We included two interaction terms in order to test a priori hypotheses regarding the effects of intraspecific and interspecific competition and how those may vary between shrimp species.

Finally, to test for an effect of shrimp size on mortality, we modeled the likelihood of mortality for those shrimp included at the outset of the experiment (generalized linear model with a binomial distribution and logit link function). Our initial logistic regression model used initial shrimp mass, density treatment, and their interaction to predict the probability of mortality, but the interaction term was not significant in either case and was removed from the final models. Due to low mortality in the overlap experiment, we constructed logistic regression models only for the brown and white shrimp monoculture experiments.

All animals sampled in the field were collected under the auspices of a South Carolina Department of Natural Resources Scientific Collecting Permit to RPD; all animals captured were immediately euthanized in an ice slurry, and no protected species were captured. Laboratory experiments were conducted under the University of South Carolina’s Institutional Animal Care and Use Committee Protocol no. 2637-101784-031623.

## Results

### Field sampling

**Fig 1:**
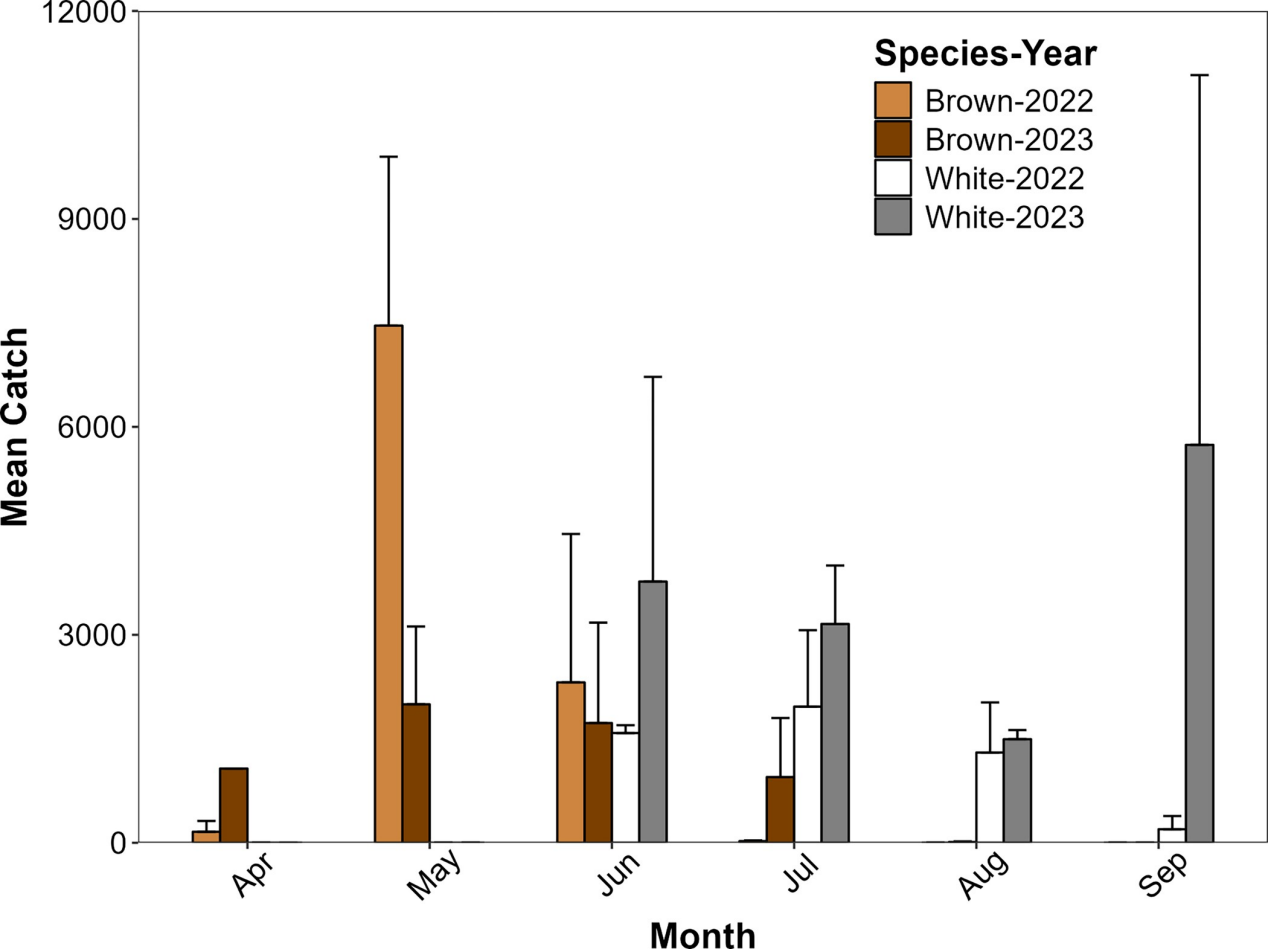
Shrimp seine catches. The relative abundance (number of individuals per seine; mean catch + SE) for April–September 2022 and 2023 of brown shrimp and white shrimp during their periods of creek habitat use derived from biweekly seine collections in Oyster Landing Creek.

Penaeid shrimp use of Oyster Landing Creek by brown and white shrimp varied considerably through time and exhibited interannual variation. In both 2022 and 2023, brown shrimp were collected in substantial numbers from April through June (mean catch of > 150 individuals per seine haul) and reached a peak in relative abundance in May (Fig 1). Average catch in July varied between the two years (mean of 23.5 individuals per haul in 2022, mean of 948 individuals per haul in 2023), and catches dropped to near zero in August and September of both years. In contrast, white shrimp were absent in seine hauls conducted in April and May but were collected in consistently high numbers June through September (mean of > 140 individuals per haul in 2022 and 2023). Based on the monthly catches, we considered June and July the primary months of temporal overlap between species. Mean relative abundance of white shrimp exceeded that of brown shrimp in both 2022 (white: 1,773 individuals; brown: 1,168) and 2023 (white: 3,523 individuals; brown: 1,415); however, we observed no evidence of a significant difference between species in either 2022 (*W* = 4, *p* = 0.343) or 2023 (*W* = 4, *p* = 0.548). Interestingly, brown shrimp were more abundant in 2022 compared with 2023, while white shrimp exhibited the opposite pattern- greater abundance in 2023 (Fig 1).

**Fig 2:**
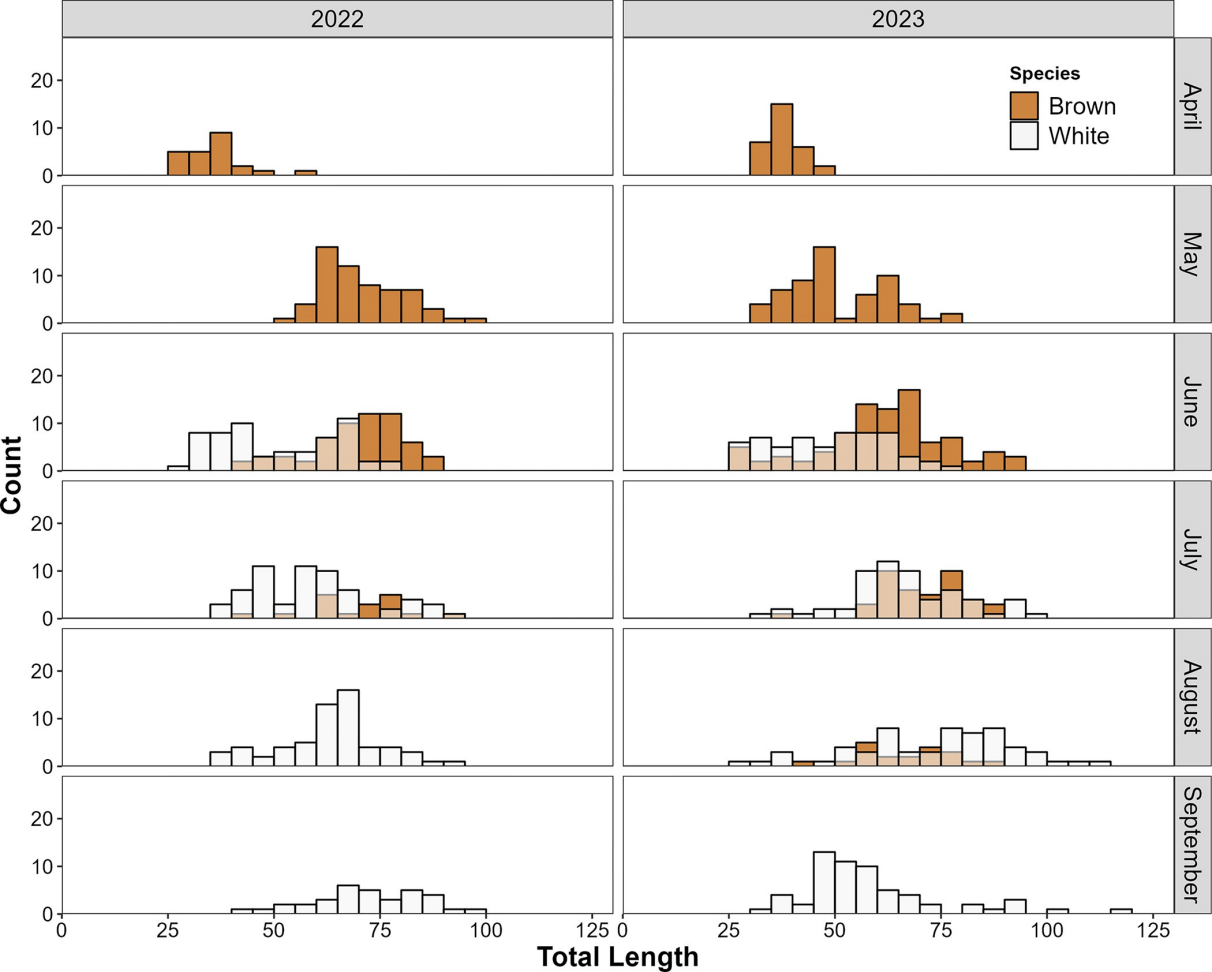
Shrimp size distribution. Monthly length-frequencies for brown shrimp and white shrimp during their periods of creek habitat use derived from biweekly seine collections in Oyster Landing Creek during April–September 2022 and 2023.

Trends in length were generally similar between species, apart from the approximately 2-month temporal offset (Fig 2 and S3 Fig in S1 File). In 2022, mean length of brown shrimp increased from initial occurrence in April (36.4 mm TL) to a maximum in May (70.4 mm TL), where it subsequently remained in both June (69.4 mm TL) and July (69.5 mm TL). Mean lengths rose more gradually in 2023, yet started (37.2 mm TL) and peaked (70.5 mm TL) at similar values. Mean length of white shrimp likewise increased during their period of estuarine residency, from a minimum in June 2022 (50.6 mm TL) and 2023 (48.2 mm TL) to a maximum in August 2022 (72.5 mm TL) and September 2023 (73.4 mm TL) (S3 Fig in S1 File). During June and July, the months of temporal overlap, the range of brown and white shrimp sizes tended to overlap in both years (Fig 2). However, results of the K-S tests provided strong evidence that brown shrimp length-frequency distributions were significantly different than those of white shrimp in both June and July of each year (2022 and 2023), leading to significantly larger brown shrimp lengths on average) across all four months of comparison (S2 Table in S1 File).

### Experiments

**Fig 3:**
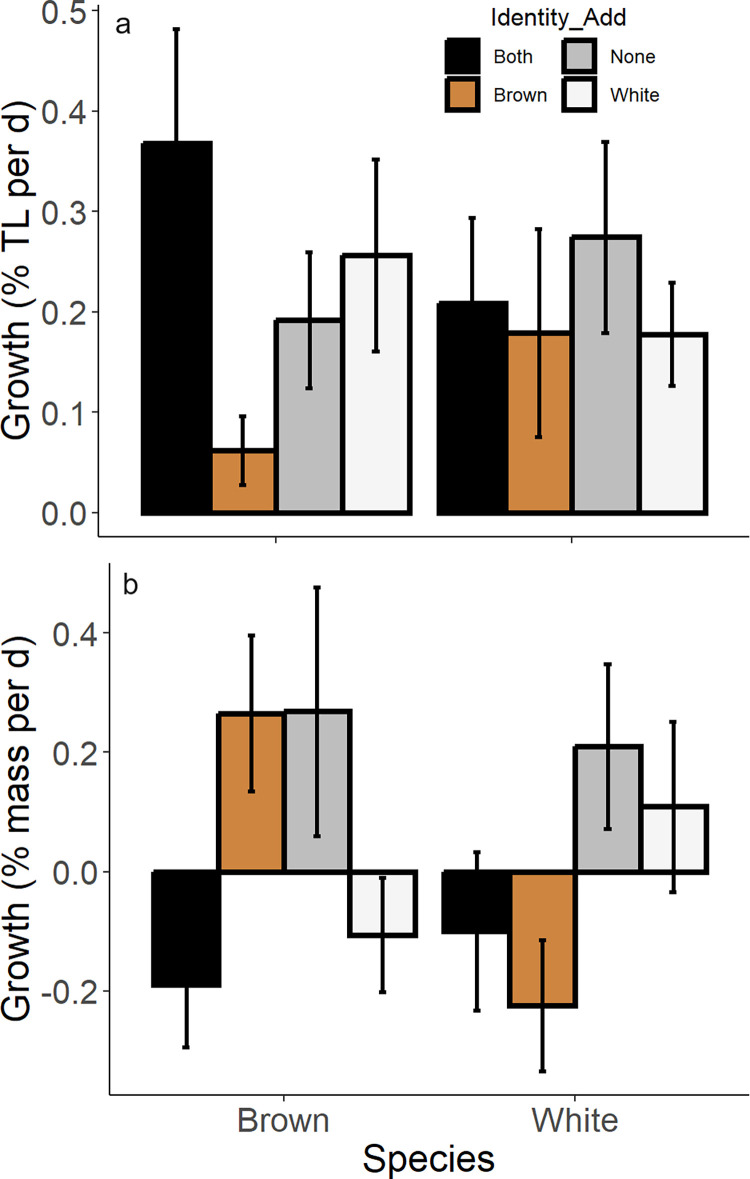
Experimental shrimp growth rates. Instantaneous growth (a- total length; b- mass) of brown shrimp and white shrimp in experiments comparing different density and species composition treatments. ‘Identity_Add’ (different colored bars) denotes the species identity of any additional shrimp found in each experimental arena.

Individual growth trajectories over 15 d for both brown and white shrimp in monoculture were variable, with some shrimp growing longer or gaining mass, some slightly losing mass, and others demonstrating no change in size, independent of density level (S4 Fig in S1 File). Instantaneous growth rates in terms of total length (% per day) were variable across species, with no clear trends based on either shrimp density or the identify of additional species (Fig 3A and S5 Fig in S1 File, Table 2). Conversely, instantaneous growth in mass (% per day) was clearly impacted by shrimp density as well as the identity of any additional shrimp (Table 2). Specifically, for both species, when either a conspecific was added or no additional shrimp were added, growth rates were positive; when both species or exclusively the other species was added, growth rates (mass % per day) were negative (Fig 3B). We observed a similar pattern in terms of density, where at low and medium densities, growth rates were positive for both species, while growth rates were negative for both species at the highest density level (S5 Fig in S1 File).

**Table 2:** Output of analysis of variance models for shrimp experiments.

	Total length				Mass				Mortality			
	Df	SS	*F*	*p*	Df	SS	*F*	*p*	Df	SS	*F*	*p*
Species	1	0.009	0.05	0.81	1	0.48	0.61	0.43	1	0.26	1.58	0.21
Density added	1	<0.001	<0.001	0.99	1	6.59	8.48	0.004	1	0.33	1.98	0.16
Identity added	3	1.18	2.30	0.08	3	2.41	1.03	0.38	3	1.64	3.24	0.023
Species * Density added	1	0.099	0.58	0.45	1	2.63	3.38	0.067	1	0.15	0.91	0.34
Species * Identity added	3	1.577	3.07	0.03	3	8.99	3.86	0.010	3	3.85	7.61	<0.001
Residuals	231	39.60			241	187.10			230	38.76		

Statistical model output for analysis of variance (ANOVA) for each of three response variables. Df denotes degrees of freedom, SS denotes sum of squares. Both length and mass were calculated as instantaneous values, with units of % d^-1^.

**Fig 4:**
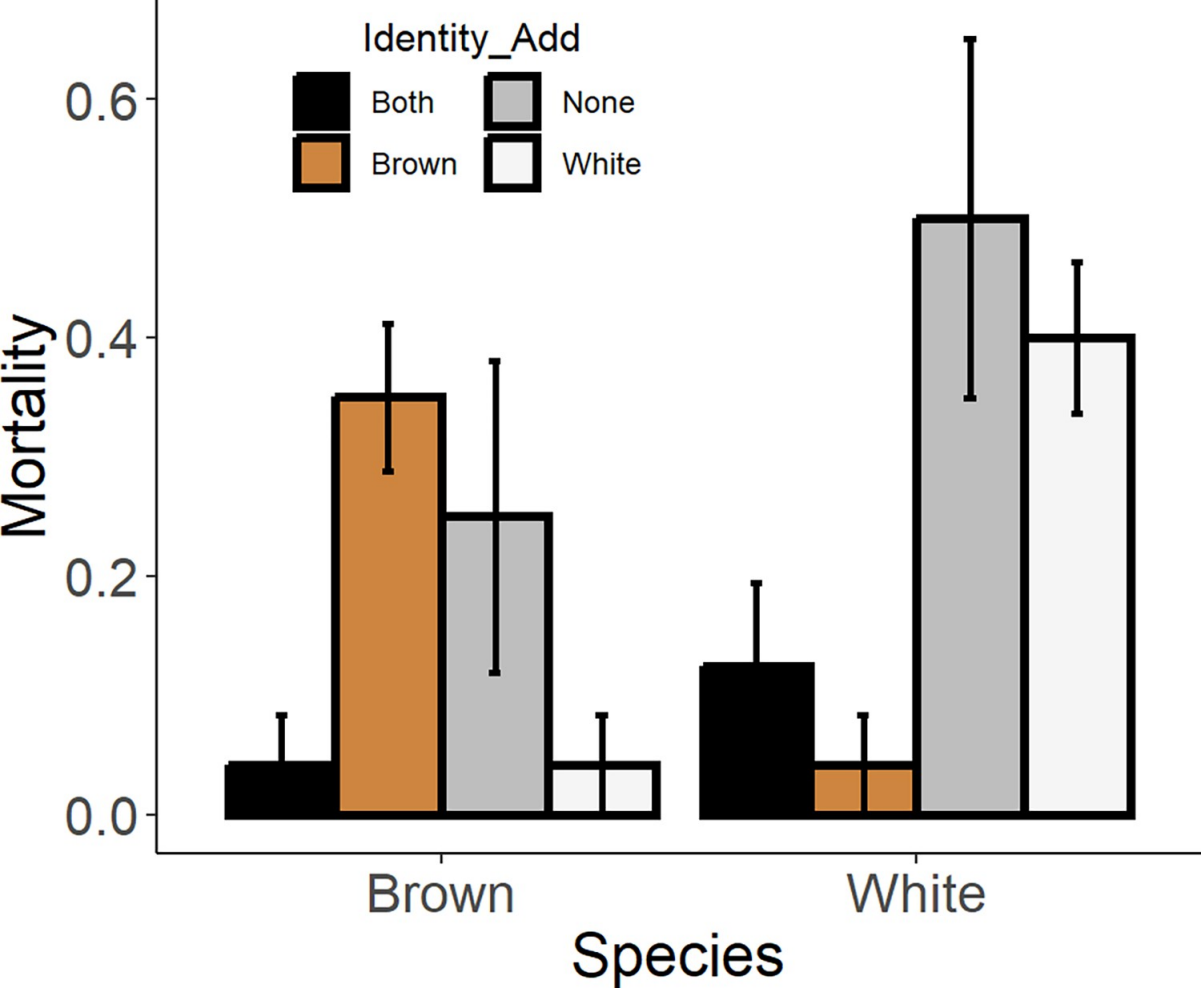
Experimental shrimp mortality rates. Mean per capita mortality of brown shrimp and white shrimp in experiments investigating density-dependence in juvenile penaeids under different density and species composition treatments.

Mortality rates were variable across species and identity added treatments (Fig 4 and Table 2). Surprisingly, mortality rates tended to be higher in the ‘none’ added and monoculture treatments for both species, while mortality rates were lower in the treatments in which ‘both’ species were added and when only the competitor species was added (Fig 4). There was no clear evidence of D-D mortality for either species (S5 Fig in S1 File, Table 2).

**Fig 5:**
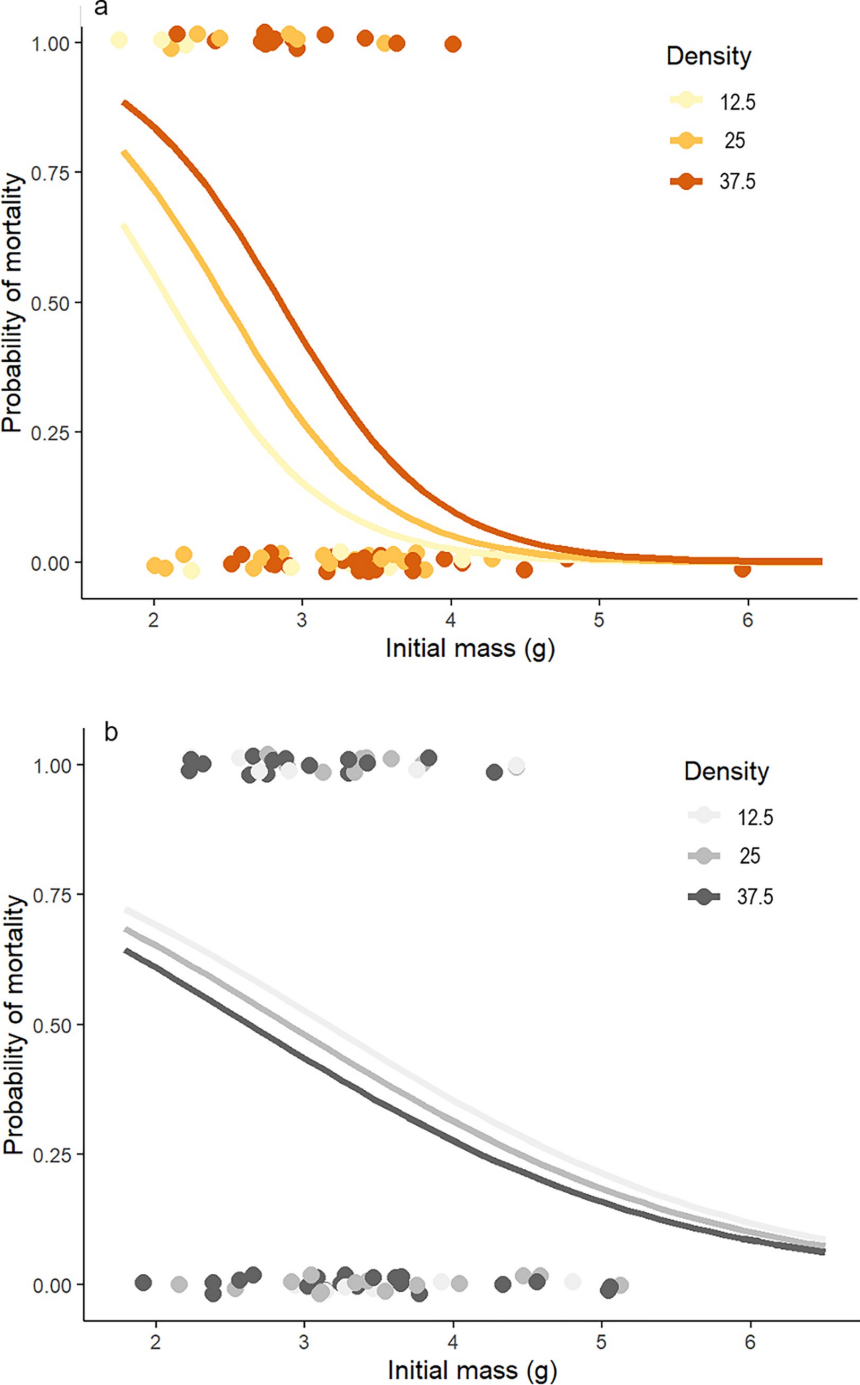
Probability of mortality across shrimp size. Probability of mortality for brown (a, n = 72) and white (b, n = 72) shrimp in monoculture as a function of initial mass, with each density treatment shown as a separate model fit. Curves represent logistic regression with a binomial distribution and a logit link function.

For the initial 72 shrimp in each monoculture trial, the probability of mortality over 15 days declined with increasing body size for both brown and white shrimp (body size factor, both *p* ≤ 0.05; Fig 5). However, the shape of that effect differed by species, with the regression coefficient for initial mass nearly 3x steeper for brown (coefficient estimate ± SE = -1.9 ± 0.56) than white shrimp (-0.7 ± 0.37). Based on logistic regression, the effect of conspecific density on the probability of mortality differed by species: brown shrimp exhibited a marginally significant trend towards D-D mortality when also accounting for shrimp size (*p* = 0.09) while white shrimp did not exhibit density-dependence (*p* = 0.58; Fig 5).

## Discussion

Temporal habitat partitioning is a critical process to facilitate species coexistence, particularly at diel scales [39,40]. Seasonal habitat utilization is an additional mechanism to facilitate persistence of spatially sympatric species over evolutionary timescales [41,42]. While teleost fishes have proven to be useful models in investigations of density-dependent (D-D) interactions and to explore the impacts of different types of competition under varying density scenarios [1,5,43–45], similar experimental tests of these processes using crustaceans are rare. Previous observational studies on penaeid shrimp have demonstrated that conspecific density and biotic and abiotic environmental conditions can contribute to the strength of density-dependence during the estuarine nursery period [2,46]. However, to our knowledge no previous manipulative experiments focusing on these species have investigated the relative importance of conspecific and congeneric abundance on vital rates.

Our field sampling documented a significant degree of temporal habitat partitioning between two congeneric species, but also a potentially consequential period of temporal overlap when large brown shrimp begin leaving tidal creeks and smaller white shrimp are recruiting. Our lab experiments designed to approximate these periods revealed high variation in individual growth trajectories during the estuarine residency period regardless of density, as well as the negative impacts of the alternate shrimp species on body mass growth rates. However, mortality rates for both species tended to be higher when alone or found in monoculture compared to treatments including congeners. Mechanistically, we speculate this could occur due to increased stress associated with being at high density among a competing species (leading to lower body mass growth), and through a reduction in cannibalism due to lower conspecific abundance (reducing mortality). Likely, these two results are linked. In monoculture, cannibalism appeared to occur immediately following ecdysis, mainly in the high density treatments. Because shrimp exhibited reduced growth rates in treatments including both species, there were fewer opportunities to consume recently molted individuals leading to lower mortality rates. Cannibalism is recognized as an important source of mortality for shrimp in both aquaculture [47] and toxicology [48] applications, but additional explorations of cannibalism as a compensatory ecological process are warranted. For example, cannibalism is considered to be a key contributor to disease outbreaks in shrimp populations via horizontal transmission [47] which should act to reduce the likelihood of both disease transmission and cannibalism due to the D-D nature of these processes. An important caveat to our results regarding tradeoffs between growth and mortality is that growth rates were calculated only for the individual shrimp which survived at least 5 days within experimental treatments. Thus, we are missing a potential source of variation in growth by excluding animals which died (potentially cannibalized) quickly. Ultimately, the emergent effects of the tradeoff between higher incidence of cannibalism and lower growth at high densities versus lower cannibalism and increased growth at low densities on shrimp metapopulations remains an intriguing question, especially considering the potential for highly connected sub-populations of penaeid shrimp species [49].

Shrimp life history occurs on an annual scale and shrimp populations exhibit high interannual variability that is likely driven by their sensitivity to factors such as habitat availability [15,36] and environmental conditions within estuaries and the coastal ocean [27,50,51]. Our field sampling reveals that juvenile brown and white shrimp generally demonstrate species-specific patterns of tidal creek nursery habitat use in a warm-temperate estuarine system. Despite significant changes in tidal creek nursery conditions over decadal scales [21–23], this habitat utilization was in line with historical patterns from this estuary. Over a nearly 20-year period spanning the 1980s and 1990s, peak abundance of both species in the same tidal creek sampled here (Oyster Landing Creek) was observed in the same months (brown shrimp: May–June, white shrimp: July–Sept) as our current study, with little apparent difference in the collected size ranges [19]. In addition, the timing of tidal creek use for these species documented in the North Inlet estuary is similar to temporal habitat utilization for penaeid shrimp from estuaries along the southeast US and Gulf of Mexico [28,36]. Thus, the patterns of density-dependence and species interactions that we explored in manipulative experiments are potentially applicable across a broad geographic distribution, from the recent penaeid shrimp expansion into Chesapeake Bay [52] to the northern Gulf of Mexico.

Across their global range, other penaeid shrimp species also exhibit significant spatial sympatry in their distributions, often accompanied by strong patterns in temporal and habitat-type partitioning [53–56]. The evolutionary emergence of these species is closely linked to variation in environmental tolerances, spawning preferences, and subsequent geographical separations linked to planetary-scale processes (e.g., sea level changes, movement of oceanic plates) [57,58]. However, the shared patterns of seasonal, species-specific nursery habitat use suggest the importance of temporal partitioning in sustaining distinct niches between otherwise similar species. Nonetheless, the habitat partitioning exhibited here by brown and white shrimp was neither exact nor absolute. Brown and white shrimp are both abundant during the months of June and July within an overlapping size range, supporting our experimental examination of interspecific (as opposed to solely intraspecific) interactions between these two species. In addition, these interactions may be intensified by changing climatic conditions and anthropogenic disturbance because of species-specific environmental preferences (as described previously).

By tagging and tracking individual shrimp within our manipulative experiments, we were able to generate size- and context-specific estimates of growth and mortality, which would not have been possible based on mean values calculated from all animals within an experimental arena. Given our experimental setup, this method increased our replication by more than 3x. However, we acknowledge that our method of tagging varied across experimental trials (PIT tags for monoculture experiments, tailfin clip for the overlap experiment) and thus our estimates of shrimp growth and mortality should be viewed considering these methodological differences. Whether the observed low mortality in the overlap experiment was due to the lack of PIT tags or the experimental treatment is unclear. However, our previous validation of the PIT tagging procedure for white shrimp demonstrated no differences in growth or mortality between PIT tagged and control, untagged individuals [34], similar to the majority of PIT tagging studies on estuarine finfish [59]. Thus, we believe the reduced growth rates and low mortality observed in treatments including both shrimp species to be a function of the imposed experimental treatments, whereby reduced growth led to fewer opportunities to cannibalize conspecifics as described above.

The species-specific growth patterns exhibited by our experimental shrimp could be underpinned by behaviors previously described for penaeids in aquaculture applications. For example, the Pacific whiteleg shrimp (*Penaeus vannamei*) can form dominance hierarchies which impact feeding and movement behaviors, and the importance of these social dynamics varies with conspecific density (i.e., dominance effects themselves are D-D) [60]. At low densities, dominant shrimp spent less time feeding, while that difference disappeared at high densities [57]. Whether brown and white shrimp exhibit similar social dynamics is unknown, but dominance hierarchies could have played a role in our experimental results. Variations in conspecific density can also lead to surprising behavioral effects which may explain the weak D-D growth observed here for white shrimp. Shrimp at low densities can exhibit increased time spent exploring and less time feeding compared to high density conditions, as well as increased per capita food consumption by shrimp at higher densities [60]. These may be the result of reduced social cues between shrimp, which can inform other individuals where prey resources are located [61]. Similarly, we utilized small absolute numbers of shrimp in each mesocosm (one to three individuals), which was due to our need to mimic naturally occurring densities while also generating sufficient replication for robust analyses. While we acknowledge that our experimental results may be impacted by either of the above mechanisms, we are unable to separate whether any particular response is a function of organismal density or absolute number. Finally, our finding of weakly negative D-D growth in white shrimp aligns with a previous aquaculture investigation, in which juvenile shrimp of a smaller size range (initial weight ~0.25 g) stocked at somewhat higher densities (28–57 m^2^) than our experiment exhibited no difference in growth rates across densities, and only at substantially higher densities did reductions in growth occur [62]. In conjunction with these results from aquaculture settings, our findings underscore the need for a mechanistic understanding of D-D interactions, whose emergent effects can exhibit substantial nuance because of organismal behavior and social dynamics.

A critical decision in our experimental design was the provision of a standardized, single food ration. However, species coexistence and reduction in inter-specific (interference) competition can be achieved through the use of different food resources, foraging behavior, or prey preferences. Both brown and white shrimp are omnivorous and can consume a wide range of prey types, including detritus and vegetation, in addition to small infauna and epifauna. Their apparent omnivory, coupled with the challenge of shrimp gut content identification, has led to contrasting evidence regarding the degree of differentiation in their diets. For example, brown shrimp may be more closely linked to infaunal populations (particularly polychaetes) than white shrimp [63]. Nonetheless, white shrimp are capable of regulating the seasonal cycles of populations of benthic infauna, including polychaetes [35]. Immunoassays of shrimp diets revealed that brown shrimp consume a wide range of macro- and meiofaunal taxa, while white shrimp consistently consumed three species of crustaceans [64]. These authors also investigated the potential for ontogenetic diet shifts in both shrimp species but found little evidence of changes in diet with size [61]. To date, ontogenetic shifts in juvenile shrimp diets, and differences between species, remain poorly understood, and given that the two species exhibited significantly different size structure during their overlap period, variation in diet with size may account for and enable coexistence. Finer scale behavioral differences and habitat preferences (e.g., burrowing, differential habitat use of marsh edge or surface, substrate preferences), likely also serve to broaden each species niche and could reduce the impact of interspecific competition.

It is important to understand the impacts of both biotic and abiotic factors on penaeid shrimp population dynamics because these two species make up the bulk of the commercial shrimp landings along the Atlantic coast of the southeastern U.S. Shrimp harvested in this region are worth tens of millions of dollars annually, but landings have been highly variable over the past two decades. Concurrently, environmental conditions within estuaries are changing, including increasing temperatures and spatially variable trends in salinity [65–67], both of which are particularly important for shrimp. The two shrimp species investigated here exhibit different salinity preferences, with brown shrimp avoiding very low salinity conditions but white shrimp demonstrating no preference [29]; similarly, brown and white shrimp postlarvae exhibit different responses to winter temperature conditions. Thus, we expect species-specific responses to future environmental conditions. Because the value of estuaries as nursery habitat for nekton varies both spatially and temporally [17] due to natural variation along estuarine gradients (e.g., salinity, productivity), we expect D-D processes such as growth and mortality will also exhibit context-dependence as estuarine nurseries experience change due to storms, sea level rise, and anthropogenic development.

## Supporting information

S1 File Supplementary information. (PDF)
