# Supplementary material for: Temporal habitat partitioning and resource competition between congenerics: Testing for density-dependent growth and mortality in estuarine shrimp
Source: PLoS One. 2024 Dec 27;19(12):e0316219. doi: 10.1371/journal.pone.0316219 (PMC11676493; doi:10.1371/journal.pone.0316219)
Supplement: S1 File — (PDF) [file pone.0316219.s001.pdf]

Supplementary Material

Temporal habitat partitioning and resource competition between congenerics: testing for density-dependent growth and mortality in estuarine shrimp

Robert P. Dunn, Matthew E. Kimball, Bruce W. Pfirrmann, Andrew S. Bruck, Willa M. Lane

Corresponding author: Robert P. Dunn, robert@baruch.sc.edu, University of South Carolina

Table S1: Statistical results of analysis of variance comparing mean growth rate of white and brown shrimp with (or without) a tailfin clip over the 14 day duration of the tailfin clip retention experiment. Response variable is growth in terms of mass (g d^{-1}). Initial lengths (mean total length in mm with one standard error) for clipped and control shrimp were not statistically compared, but were 64.8 (3.28) and 74.4 (3.6) for brown shrimp, and 72.8 (3.2) and 55.8 (3.34) for white shrimp (clipped and control, respectively). No mortalities were observed in either clipped or control shrimp of either species over the 14-d experiment. All tailfin clips remained visible with little to no regeneration during the experimental period.

	**DF**	**Mean Square**	***F***	***p***
Species	1	< 0.001	< 0.001	0.99
Tailfin clip	1	< 0.001	< 0.001	0.99
Species x Tailfin clip	1	< 0.001	0.06	0.80
Residual	15	0.013		

Table S2: Mean (SE) total length (mm) of brown and white shrimp during their identified months of temporal overlap within Oyster Landing Creek in 2022 and 2023, and results of Kolmogorov-Smirnov tests comparing size distributions between species. **Bold** indicates statistically significant comparisons ($p < 0.05$)

**Month - Year**	**Shrimp Total Length**		**K-S Test *U***	***p***
	**Brown**	**White**		
June 2022	69.4 (1.4)	50.6 (1.8)	0.583	**< 0.001**
July 2022	69.5 (2.7)	58.6 (1.9)	0.556	**< 0.001**
June 2023	61.4 (1.6)	48.2 (1.7)	0.433	**< 0.001**
July 2023	70.5 (1.6)	66.4 (1.9)	0.338	**0.003**

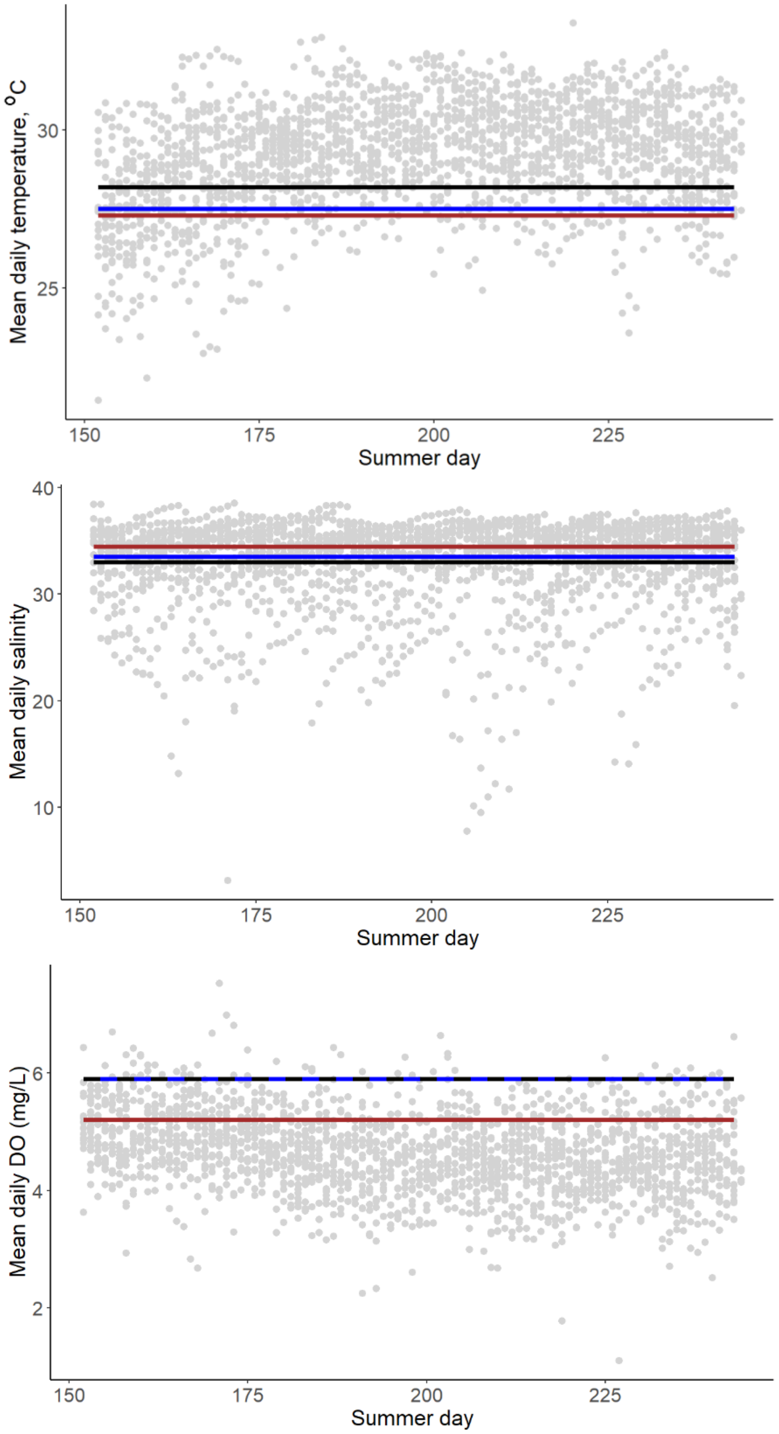

Figure S1: Mean environmental conditions within the N = 36 mesocosms in our three experiments shown with horizontal lines (brown = brown shrimp, black = white shrimp, blue = overlap). Experimental data are overlaid on daily means for summer daylight hours over the period 2001 - 2023 collected in situ at the Oyster Landing System-Wide Monitoring Program station. This site represents typical summertime conditions in tidal creeks within the North Inlet estuary.

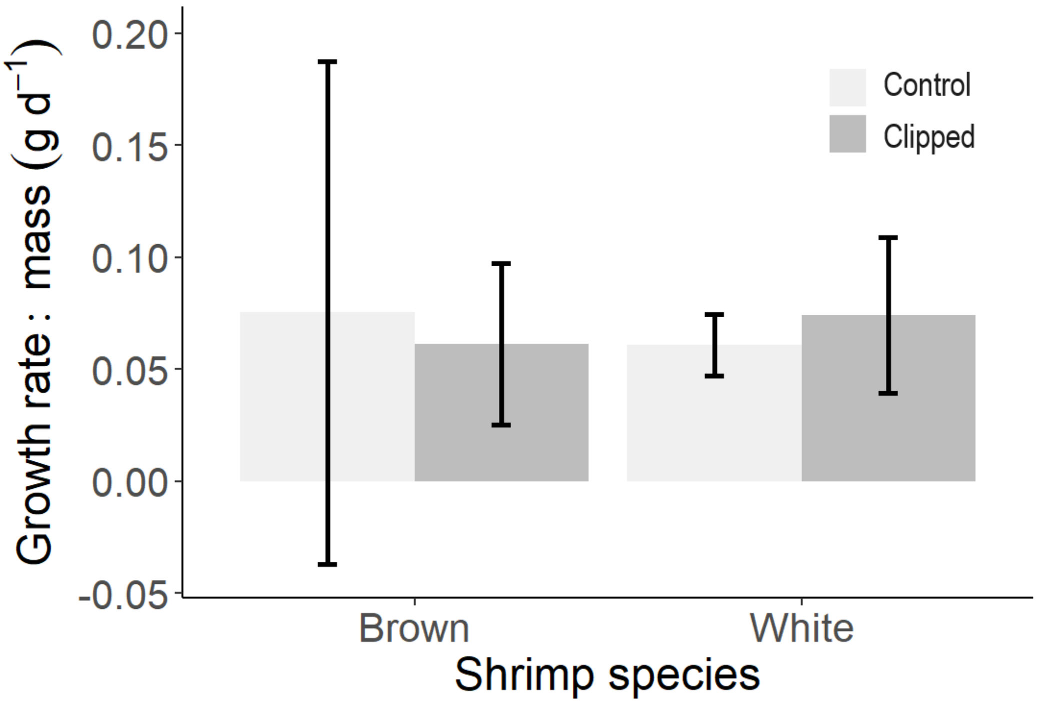

Figure S2: Results of tailfin clip retention experiment for brown and white shrimp conducted in the Baruch Marine Field Laboratory flow-through seawater facility during July 2023. No mortalities were observed in either clipped or control shrimp of either species over the 14-d experiment. All tailfin clips remained visible with little to no regeneration during the experimental period.

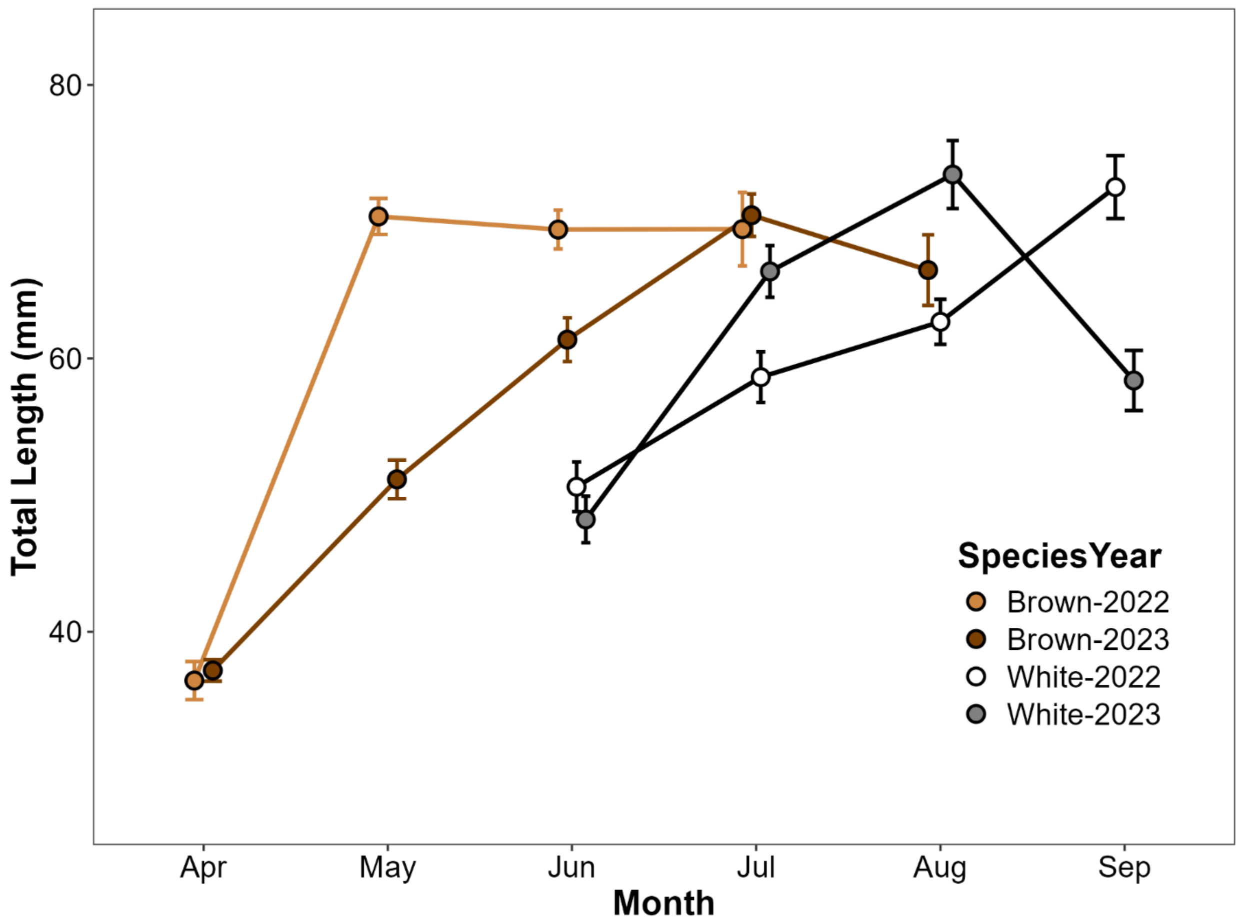

Figure S3: Monthly total length (mm ± SE) of brown and white shrimp collected in Oyster Landing Creek from April to September (2022 and 2023 combined).

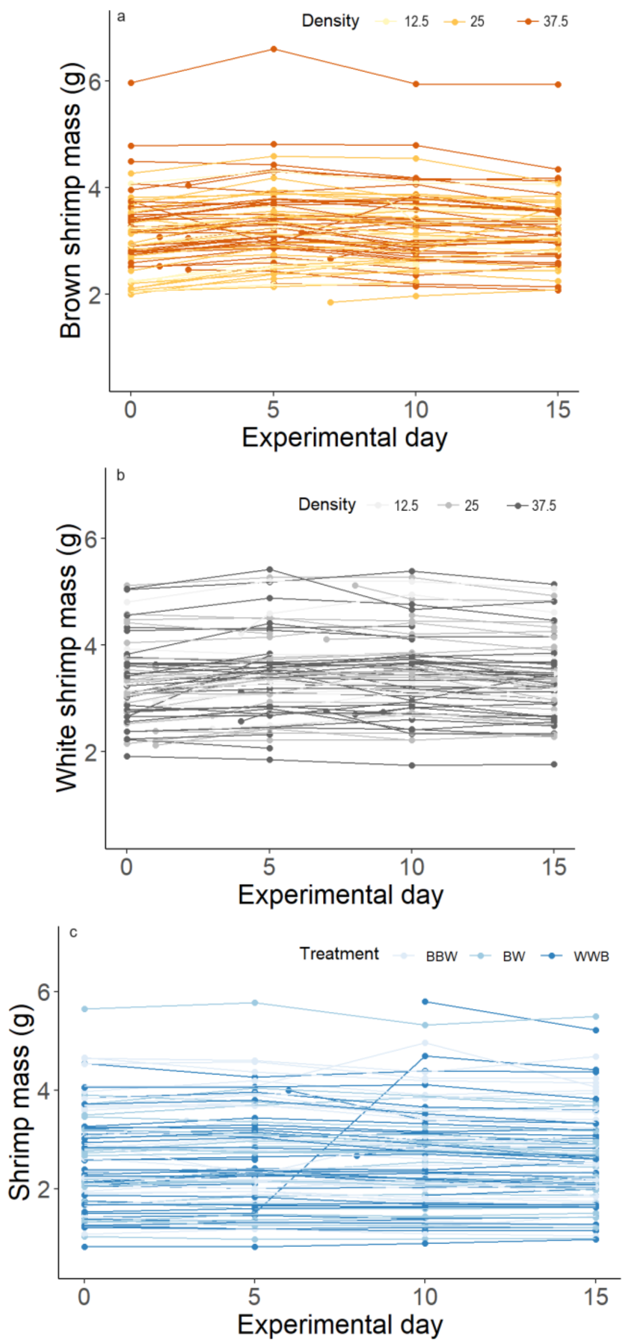

Figure S4: Individual growth trajectories, in terms of mass, for brown and white shrimp in monoculture (a, b respectively) and during the overlap trial with both species together (c) during experiments conducted in flow-through seawater laboratory mesocosms. In the top two panels, lines are colored according to density treatment. In the bottom panel, lines are colored by species composition treatment (BBW = 2 brown, 1 white; BW = 1 brown, 1 white; WWB = 2 white, 1 brown; corresponding densities: BW = 25 m-2, BBW & WWB = 37.5 m-2). Shrimp were individually identified with either PIT tags or tailfin clips. Only animals that survived for ≥ 5 days are shown, and animals that entered the experiment on non-standard sampling days (0, 5, 10) were replacements.

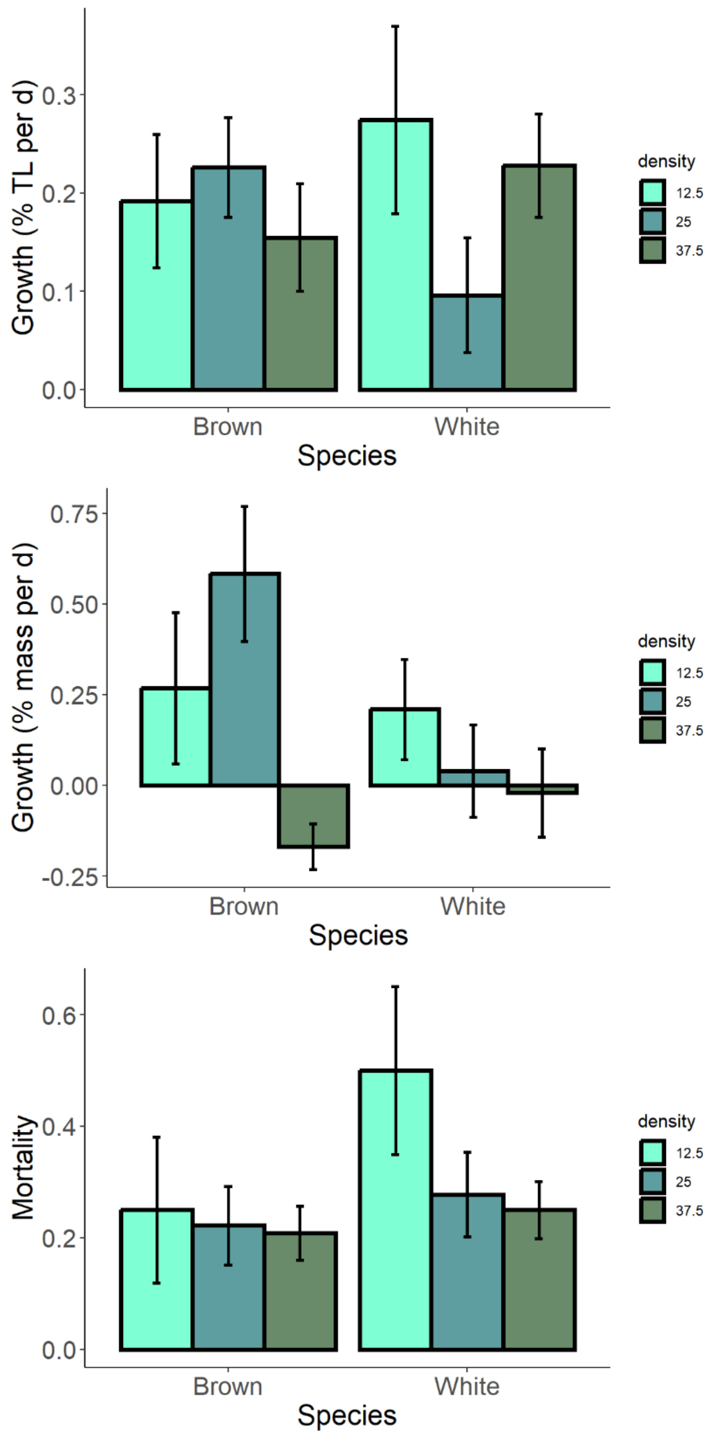

Figure S5: Instantaneous growth (length and mass) and per capita mortality showing species-specific rates across each of the three density treatments.
